# A nearly gapless, highly contiguous reference genome for a doubled haploid line of *Populus ussuriensis*, enabling advanced genomic studies

**DOI:** 10.48130/forres-0024-0016

**Published:** 2024-05-13

**Authors:** Wenxuan Liu, Caixia Liu, Song Chen, Meng Wang, Xinyu Wang, Yue Yu, Ronald R. Sederoff, Hairong Wei, Xiangling You, Guanzheng Qu, Su Chen

**Affiliations:** 1 State Key Laboratory of Tree Genetics and Breeding, Northeast Forestry University, Harbin 150040, China; 2 College of Life Science, Northeast Forestry University, Harbin 150040, China; 3 Forest Biotechnology Group, Department of Forestry and Environmental Resources, North Carolina State University, Raleigh, NC 27695, USA; 4 College of Forest Resources and Environmental Science, Michigan Technological University, MI 49931, USA; 5 Key Laboratory of Saline-Alkali Vegetation Ecology Restoration, Ministry of Education, Northeast Forestry University, Harbin 150040, China

**Keywords:** Doubled haploid, Genome assembly, Telomere, Centromere, *Populus ussuriensis*, T2T genome

## Abstract

*Populus* species, particularly *P. trichocarpa*, have long served as model trees for genomics research, owing to fully sequenced genomes. However, the high heterozygosity, and the presence of repetitive regions, including centromeres and ribosomal RNA gene clusters, have left 59 unresolved gaps, accounting for approximately 3.32% of the *P. trichocarpa* genome. In this study, the callus induction method was improved to derive a doubled haploid (DH) callus line from *P. ussuriensis* anthers. Leveraging long-read sequencing, we successfully assembled a nearly gap-free, telomere-to-telomere (T2T) *P. ussuriensis* genome spanning 412.13 Mb. This genome assembly contains only seven gaps and has a contig N50 length of 19.50 Mb. Annotation revealed 34,953 protein-coding genes in this genome, which is 465 more than that of *P. trichocarpa*. Notably, centromeric regions are characterized by higher-order repeats, we identified and annotated centromere regions in all DH genome chromosomes, a first for poplars. The derived DH genome exhibits high collinearity with *P. trichocarpa* and significantly fills gaps present in the latter's genome. This T2T *P. ussuriensis* reference genome will not only enhance our understanding of genome structure, and functions within the poplar genus but also provides valuable resources for poplar genomic and evolutionary studies.

## Introduction

Poplars, fast growing tree species with relatively short life cycle, are widely distributed across northern temperature regions, spanning from North America through Eurasia to Northern Africa^[1]^. Their versatility extends beyond being used for making paper, pallets, furniture, and kitchen supplies. They are also highly suitable for reforestation due to their pioneer tree species characteristics^[2]^. Poplars are known for their ability to produce large quantities of seeds and their roots readily sprout on marginal lands^[3]^. Due to its modest genome size, rapid growth rate, facile vegetative propagation methods and high amenability for genetic manipulation, *Populus* has emerged as the model species for genetic and molecular studies of forest trees in the realm of forest trees^[4]^.

*Populus trichocarpa* is the first tree species and the third plant species to have its whole genome sequenced^[5]^, four years after *Arabidopsis thaliana* genome^[6,7]^ and one year after *Oryza sativa* genome^[8]^ was sequenced. In recent years, several poplar genomes including *P. alba*^[9]^, *P. euphratica*^[10]^, *P. tremula*^[11]^, *P. ilicifolia*^[12]^, *P. pruinosa*^[13]^ and *P. tomentosa*^[14]^ and a few hybrid poplar genomes including *P. alba *× *P. glandulosa*^[15]^ and *P. alba* '*Berolinensis*'^[16]^ have been sequenced. However, owing to the high heterozygosity and highly repetitive sequences present in the genomes, these published genome assemblies are not highly contiguous, and incomplete in the repetitive regions, centromeres and telomeres^[17]^.

Poplars are dioecious plants, characterized by highly heterozygous genome^[1]^. These genomes have been shaped by events like whole genome duplications, widespread repetitive sequence expansions, and subsequent chromosome rearrangements, which resulted in genomes endowed with complex characteristics, and difficulty to assemble^[18]^. The pronounced genomic heterozygosity complicates efforts to achieve high-contiguity genomes, while the abundance of repetitive sequences often results in assembly gaps, particularly when using short sequence reads for diploid genome assembly. This is because biparental allelic sequences from two homologous chromosomes may be erroneously fused during assembly, leading to inaccurate gene annotation^[1]^. In the last few years, the advent of long high-throughput sequencing technologies has largely alleviated the challenges in assembling the highly repetitive regions. Nevertheless, the issue of high genomic heterozygosity remains, and the adoption of homozygous lines offers a radical solution to this challenge. Generation of homozygous lines in annual plants can take many generations, for instance, the highly homozygous cultivar PN40024 grapevine^[19]^, was developed through nine generations of selfing. This approach is not feasible for woody plant species, primarily due to their long juvenile periods. In the case of dioecious poplars, this poses a persistent challenge, as decreased heterozygosity can affect their environmental adaptability. Nonetheless, there may be potential for the induction of haploid plants and the development of homozygous diploids, albeit with considerable challenges. For instance, haploid cells derived from a single pollen grain and doubled artificially to form homozygous diploids, generally referred to as doubled haploid (DH) lines have been reported^[20]^. DH individuals possess two identical homologous chromosomes, making them ideal materials for genome sequencing. DH lines with whole genome sequencing has been reported in crops, including maize^[21]^, tomato^[22]^, barley^[23]^, *Brassica oleracea*^[24]^. However, the occurrence of haploid or DH lines in forest trees has been less frequently reported.

In this study, DH callus lines of *P. ussuriensis* were obtained through *in vitro* anther induction, and a DH line named DH15 was selected for DNA sequencing using the PacBio High Fidelity (HiFi) long-read sequencing, Illumina sequencing, and high-throughput chromosome conformation capture (Hi-C) sequencing methods. A *de novo* assembly was then performed by a combination of PacBio long reads, Illumina, and Hi-C sequencing reads, which resulted in a T2T high-quality poplar genome. This new assembly was annotated and 465 more genes were identified than that of the current v4.1version of *P. trichocarpa* genome. In the previous poplar genome assembly, the centromeres and telomeres were not at all or only partially assembled and thus not reported. A comprehensive analysis of the structures, features, composition, and distribution of these regions were conducted, successfully closing nearly all the gaps in the newly assembled reference genomes. The structural components and characteristics of the centromeres of all chromosomes in the DH15 poplar genome were dissected and carefully annotated. Additionally, the annotation of transposable elements (TEs) and new genes in highly repetitive regions, particularly centromeres, have been improved. This refined genome assembly will be highly instrumental in molecular analyses of gene functions in poplar trees and enable comparative genomic studies across different poplar species. It serves as a solid foundation for future research on the poplar and other plant genomes.

## Materials and methods

### Plant materials and haploid calli induction

Male flower buds from a *Populus* *ussuriensis* tree were collected for anther culture at mid- or late-uninucleate stage of microspore development. Anther culture was conducted using Murashige and Skoog (MS) basal medium containing 2.0 mg/L 2,4-Dichlorophe-noxyacetic acid (2,4-D), 1.0 mg/L Kinetin (KT), 3 g/L Gelrite, and 3% sucrose to induce haploid formation. Following an initial culture in the dark for 40 d, a cold treatment of 4 °C was administered for 24 or 48 h. The anthers were continued to culture on the medium for six months. Flow cytometry was used to determine the ploidy levels of calli at different stages. Heterozygous genomic sites of the paternal *P.* *ussuriensis* tree were identified by genome resequencing. Polymerase Chain Reaction (PCR) was used to amplify ten selected heterozygous sites and then sequenced by Sanger sequencing.

### DNA extraction, library construction and sequencing

A doubled haploid line (DH15) of *P. ussuriensis* was used for genome sequencing. Genomic DNA was isolated using SMRTbell Template Prep Kit 1.0 (Pacific Biosciences, Memlo Park, CA, USA). The quality of DNA assessed by agarose gel electrophoresis and the quantity was determined using a NanoDrop spectrophotometer (Thermo Fisher Scientific). The DNA libraries were constructed as described in a previous study^[25]^. For sequencing, both Illumina and PacBio Sequel II sequencing platforms were employed. Illumina reads were utilized for genomic survey purposes, while HiFi reads from PacBio Sequel II were employed for the genome assembly.

The construction and sequencing of Hi-C library was done by Annoroad Gene Technology Company as follows: (1) DH15 calli were treated with 1% (vol/vol) formaldehyde to cross-linked DNA; (2) the cross-linked DNA was then lysed, and digested with MboI enzymes overnight; (3) the MboI enzymes were inactivated, and cohesive ends were filled in by introducing biotin-labeled dCTP; (4) after proximity ligation was performed in a blunt-end ligation buffer, the cross-linking was reversed, and DNA was purified for Hi-C library construction^[26]^; (5) the final Hi-C library was sequenced on an Illumina HiSeq 2500 platform in 150-bp paired-end mode.

### Genome assembly and assessment

The genomic size of the DH15 was estimated based on *K*-mer (k = 21) analysis using short reads, which were sequenced on the Illumina platform. The filtered PacBio HiFi reads (longer than 1,000 bp) was assembled into contigs using Hifiasm with default parameters^[27]^.

The contig-level assembly was indexed with bwa index (with -a bwtsw) (v.0.7.15-r1140) and samtools faidx. The DH15 Hi-C read pairs were aligned using bwa aln and bwa sampe. Aligned reads (in pairs) were converted into BAM files using samtools view with options of -b -F12. The BAM files were filtered with filterBAM_forHiC.pl (from ALLHiC package, v.0.9.13)^[28]^ to remove nonuniquely mapped reads. Then, for the BAM files, ALLHiC_partition was run with -e GATC -k 1 -m 25; allhic extract was run with --RE GATC option; allhic optimize and ALLHiC_build was run with default settings; the chromosome contact map was visualized with ALLHiC_plot at 100-kb resolution.

The completeness of the genome was assessed using BUSCO v.4.0.6, which contained 1,614 genes in the 'embryophyta_odb10' dataset^[29]^, with default parameters.

### Genome annotation

The repetitive sequences in the DH genome were identified as follows. Tandem repeats were identified using theTRF tool with default settings. RepeatModeler (version 2.0.1)^[30]^ was used for *de novo* identification of the repetitive sequences and RepeatMasker (version 4.1.0)^[31]^ was used to predict TE sequences based on sequence homology. Long terminal sequence repeats were identified by LTR_FINDER (version 1.1)^[32]^. RepeatClassifier^[33]^ was used to classify the identified repetitive sequences in the DH genome.

The protein-encoding genes of the DH15 genome were predicted by the combination of *de novo*, homology-based and RNA-seq data-aided methods. The AUGUSTUS model was trained and optimized using the single copy gene identified by BUSCO, and then used for *de novo* prediction. The protein sequences of ten species, *P. bolleana*, *P. tomentosa*, *P. tremula*, *P. deltoides*, *P. simonii*, *P. trichocarpa*, *P. wilsonii*, *P. euphratica*, *P. pruinose*, and *P. ilicifolia*, were used for homology-based annotation. To perform RNA-Seq assisted gene prediction, we downloaded poplar transcriptome data from the NCBI SRA database (BioProject: PRJNA808967). Clean reads were assembled into transcripts using Trinity^[34]^, which were aligned to the genome assembly using the Program to Assemble Spliced Alignments^[35]^ to predict gene structures. Finally, Evidence Modeler^[36]^ was used to combine gene annotation results from all homologous, *de novo*, and transcriptome-based predictions to integrate into a non-redundant, more complete set of genes.

Telomeric and centromeric regions of the DH15 genome were identified using quarTeT^[37]^. The TeloExplorer module in quarTeT was used to identify the telomeres in the genome, and the 'explore' and 'search' tools from the telomere identification toolkit (tidk) (https://github.com/tolkit/telomeric-identifier/) were employed by this module. And telomeres in the genome were further manually validated. The CentroMiner module makes predictions about the centromeres of the genome. Using the FASTA format of the genome as an input file and inputting the transposable element (TE) annotation in GFF3 format achieve better consequences. We then used HiCAT to annotate the centromeres of the DH15 genome with default parameters^[38]^. HiCAT takes a monomer template and a centromere DNA sequence as inputs.

### Analysis of genomic evolution and WGD events

Protein sequences of 16 plant species, *P. trichocarpa*, *P. deltoides*, *P. simonii*, *P. wilsonii*, *P. tremula*, *P. tomentosa*, *P. bolleana*, *P. alba*, *P. pruinosa*, *P. euphratica*, *P. ilicifolia*, *Salix brachista*, *S. purpurea*, *Arabidopsis thaliana*, *Carica papaya*, and *Vitis vinifera* were used to construct a phylogenetic tree. Genes with internal stop codons, incompatible reading frames, or fewer than 50 amino acids were removed. For genes with alternative splicing variants, the longest transcript was selected. Then the comparison was performed using BLASTP with an e-value cut-off of 1e-5. OrthoFinder^[39]^ was used for gene family analysis. The gene families with only one copy from each of 16 species were selected as single-copy genes and were used for subsequent analysis.

MAFFT software (v7.158b)^[40]^ was employed to generate multiple sequence alignments of protein-coding sequences for each single-copy gene. Subsequently, the alignments of all single-copy genes were concatenated to construct a phylogenetic tree using RAxML v8.2.8 software^[41]^ with 1,000 bootstrap replicates. Next, r8s software (v1.71)^[42]^ with default parameters was applied to estimate the divergence time among species. The divergence time of the existing fossil record of the *Populus* and *Salix* (48 Mya)^[43]^ was used for the phylogenetic analysis. We also based the calibration point for this estimation was based on the divergence time of *V. vinifera* and *A. thaliana* (109.8−124.4 Mya) obtained from the TimeTree (www.timetree.org). The final phylogenetic tree was visualized using the iqtree tool^[44]^. *Ks* values for each gene pair were calculated *via* KaKs_Calculator^[45]^. The distributions of all *Ks* values were plotted *via* the R software and ggplot2 package^[46]^.

### Collinearity analysis

The genomes were compared using Nucmer with the parameters '-c 100 -b 500 -l 50'^[47]^. Subsequently, the results from the alignment file generated by Nucmer were filtered using Delta-filter with parameters '-i 90 -l 100'. SyRI, a tool for identifying synteny and rearrangement^[48]^, was then used to compare the genome assemblies of chromosomes of DH15 and *P. trichocarpa* and identified syntenies and structural rearrangements. Finally, the results were visualized using Plotsr^[49]^.

## Results

### Generation of haploid and doubled haploid (DH) calli for *P. ussuriensis*

**Figure 1:**
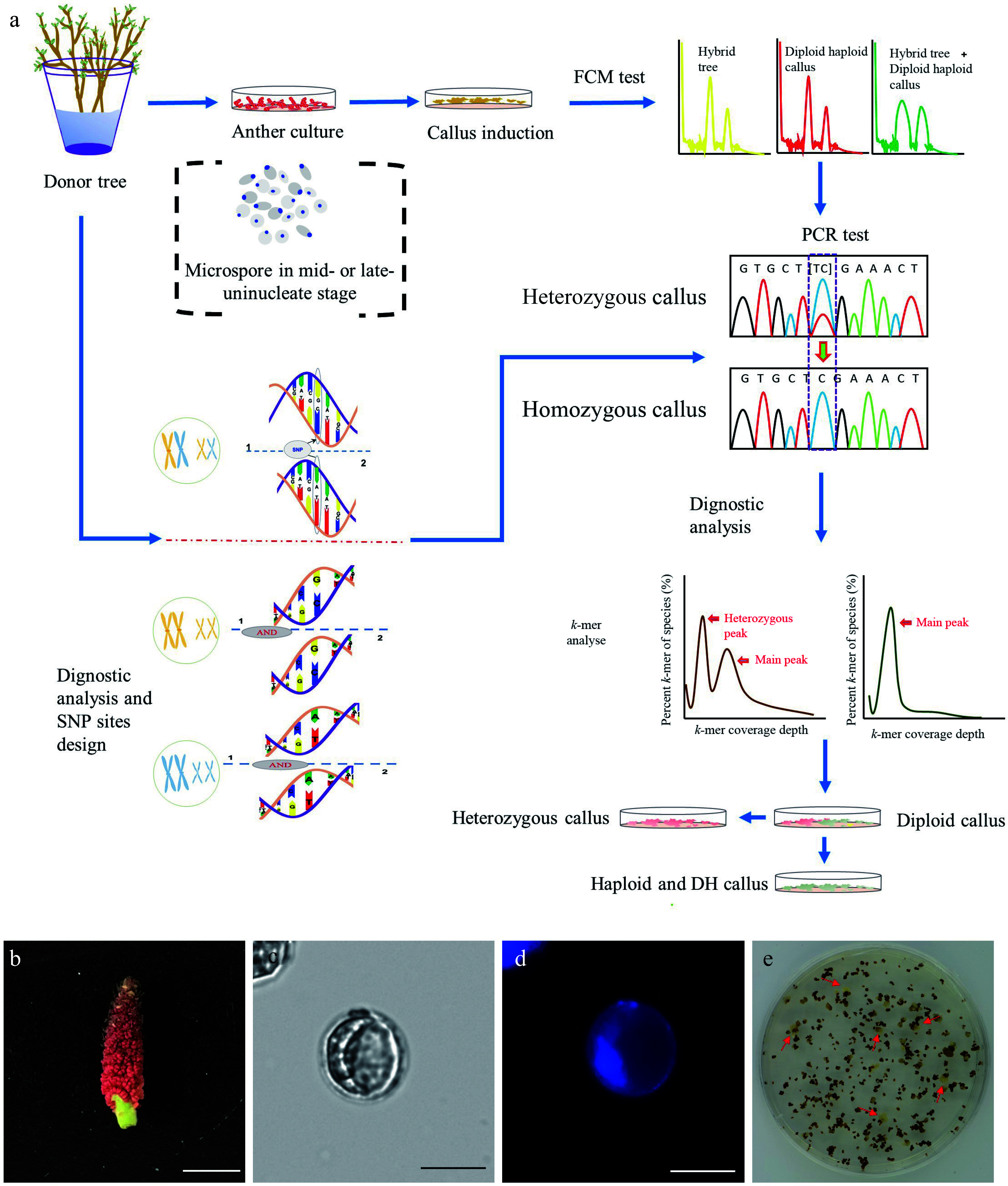
Induction of doubled haploid (DH) callus lines. (a) Flowchart illustrating the procedure of the DH callus induction and characterization. (b) Floral stage of male catkin used for anther culture. Bar = 1 cm. (c) Mid-to-late uninucleate microspores (unstaining). Bar = 25 μm. (d) DAPI staining of mid-to-late uninucleate microspores. Bar = 25 μm. (e) Callui induced from the anthers that were cultured on the callus induction medium.

*In vitro* haploid callus induction was conducted using the *P. ussuriensis* anthers collected from hydroponic branches following a procedure as shown in Fig 1a. The microspores-bearing anthers that were cytologically characterized as the mid-to-late uninucleate stage anthers were collected (Fig. 1b−d). After five weeks of culture on the induction medium, the calli that emerged from the anthers (Fig 1e) were subjected to an established high-throughput screening method to identify haploid and auto-doubled haploid calli (Fig. 1a). A whole-genome resequencing with 150 times coverage was conducted with the paternal (anther donor) tree to identify SNPs. The resulting reads were aligned to the *P. trichocarpa* genome and the Genome Analysis Toolkit (GATK)^[50]^ was used to identify SNPs, from which ten highly confident heterozygous sites were then selected. We designed ten pairs of primers based on the sequences at these selected sites (Supplemental Table S1). These primers were used for polymerase chain reaction (PCR) amplification using genomic DNA extracted from both the paternal tree and the induced calli. Only the calli that exhibited homozygosity at all ten sites were classified as haploid or DH genotypes (Fig. 1a).

A putative DH line, DH15, was eventually selected for further investigation. *k*-mer analysis was conducted with a *k*-mer size of 21 using Illumina sequencing reads totaling 64.91 Gb (Supplemental Table S2). The *k*-mer distribution of DH15 revealed a distinctive primary peak, indicative of its homozygous genomic origin (Supplemental Fig. S1a). Based on the number of the total *k*-mer and the depth of the main peak, the genomic size of DH15 was estimated as 418.47 Mb (Supplemental Table S3). In contrast, the *k*-mer distribution of the diploid paternal plant displayed the typically bimodal pattern consistent with the heterozygous genome of diploid plants (Supplemental Fig. S1b). The heterozygosity of the paternal plant was determined to be 0.71%.

### Generation of a telomere-to-telomere gap-free reference genome for *P. ussuriensis*

A total of 21.44 Gb (21,439,216,780 bp, ~50 × coverage) PacBio HiFi reads with an N50 length of 17.69 kb was generated to assemble the genome of DH15 (Supplemental Table S4). Initially, Hifiasm^[27]^ was used to assemble the HiFi reads into contigs, and a total of 706 contigs were obtained. After filtering the mitochondrial and chloroplast genome sequences out, 67 contigs were obtained with a total length of 417.48 Mb, closely matching the genomic size estimated by *k*-mer (418.47 Mb). This suggests that all the genome sequences might be assembled. Out of these contigs, 14 were identified to contain canonical telomeric repeats at both ends, indicating that these 14 chromosomes were fully assembled. Additionally, 130.65 Gb Hi-C data was generated, which measures physical associations of DNA fragments physically associate in three-dimensional space (Supplemental Table S5). This Hi-C data aided in anchoring the remaining contigs onto chromosomes. The remaining five chromosomes of the DH15 genome were further assembled with the 12 contigs. There were, however, 41 contigs totaling 5.35 Mb in length, representing approximately 1.28% of all the contigs length, which could not be anchored onto specific chromosomes.

**Figure 2:**
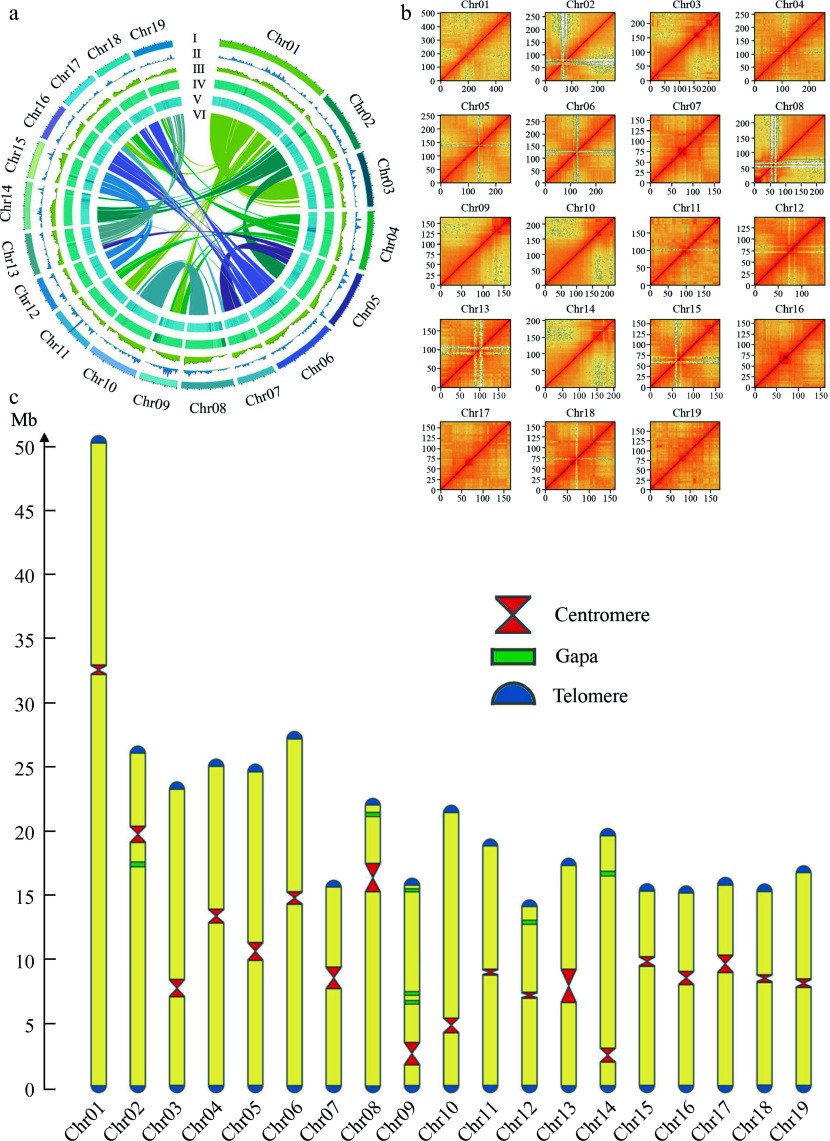
Chromosomal features of the DH15 genome. (a) Circus diagram of *P. ussuriensis* DH15 genome. Genome elements are shown in the following scheme (from outer to inner). (I) Chromosome karyotype analysis; (II) Distribution of tRNA (window size, 500 kb); (III) Gene density (window size, 500 kb). (IV) Distribution of GC content (window size, 500 kb); (V) Distribution of transposable element (TE); (VI) Syntenic relationships among different chromosomes of *P. ussuriensis*. (b) Hi-C interaction heatmap based on the chromosome-scale assembly. The map represents the contact matrices generated by aligning the Hi-C data to the chromosome-scale assembly. Heatmap shows Hi-C interactions under the resolution of 100 kb. (c) Visualization of telomeres, centromeres, and gaps position on chromosomes of *P. ussuriensis*.

The final assembly had a total length of 412.13 Mb with a contig N50 length of 19.50 Mb. The longest contig, at 50.54 Mb, represented Chr 1 of the DH15 genome (Fig. 2a). Subsequently, we conducted a comprehensive analysis, including chromosome karyotype, tRNA distribution, gene density, GC content, TE distribution, and inter-chromosomal collinearity (Fig. 2a). The 41 contigs that couldn't be anchored to the chromosomes were annotated using NCBI database and found that all these contigs corresponded to small subunit ribosomal RNA genes (Supplemental Table S6). Ultimately 19 scaffolds were acquired representing the 19 chromosomes of DH15 genome. Notably, the assembly had seven remaining gaps (Fig. 2b & Supplemental Fig. S2). These gaps were situated on chromosomes Chr 2, 8, 9, 12, and 14, with Chr 9 containing three gaps, while the others had one each. The lengths and positions of centromeres and the gap locations on chromosomes were visualized (Fig. 2c). The DH15 genome we generated exceeds the current 4.1 version assembly of *P. trichicarpa* by 22.92 Mb (Supplemental Table S7).

To assess the completeness of the assembled DH15 genome, Illumina short reads were aligned, specifically generated for the genome survey purpose, to the DH15 assembly. Remarkably, a staggering 99.88% of the short reads were successfully mapped to the contigs, with 98.37% of these mapped reads exhibiting proper pair-end mapping (Supplemental Table S8). Benchmarking Universal Single-Copy Orthologs (BUSCO) were also used to assess the genome assembly's completeness. The results revealed that 98.7% of BUSCO genes were fully covered, with an additional 0.5% being partially covered by the genome (Supplemental Table S9). All these findings collectively demonstrated the exceptionally high quality of the DH15 genome.

### Telomere and centromere structures of the DH15 genome

Telomeres, composed of highly repetitive DNA sequences, are situated at both ends of chromosomes and serve to safeguard chromosomes from degradation, repair, unwanted recombination, and fusion events^[51]^. In plants, telomere sequences are remarkably conserved, featuring a tandem repeat of unique seven-nucleotide sequence (CCCTAAA or TTTAGGG). It was found that all the 38 telomeres of the 19 chromosomes in the DH15 genome were successfully assembled. The lengths of the assembled telomeres ranged from 2,040 bp, approximately 292 tandem repeats of CCCTAAA in Chromosome 3, to 25,738 bp, approximately 3677 tandem repeats of CCCTAAA in Chromosome 14 (Supplemental Table S10). Interestingly, the two telomeres at the two ends of the same chromosome exhibited distinct lengths. For instance, the telomeres located at the five- (5') and three-prime (3') ends of the Chr14 measured 25,738 bp (the longest), approximately 3,677 tandem repeats of CCCTAAA, and 14,917 bp, approximately 2,131 tandem repeats of TTTAGGG, respectively. Across all 19 chromosomes, the average length of telomeres at the five-prime ends (5') was 12,891 bp, which is roughly equivalent to 1,842 tandem repeats of CCCTAAA. In contrast, the telomeres at the three-prime (3') ends have an average length of approximately 14,430 bp, corresponding to approximately 2,061 tandem repeats of TTTAGGG. The median lengths at the five-prime ends (5') were 13,093 bp, which is approximately equivalent to 1,870 tandem repeats of CCCTAAA. In contrast, the median length at the three-prime (3') ends was approximately 14,917 bp, which corresponds to roughly 2,131 tandem repeats of TTTAGGG. The telomere at three-prime end (3') of Chr19 was notably 4.25 times longer than that at its five-primer end (5'). The reasons behind these variations, at both cytological and molecular levels, remain unclear. It's worth noting that the DH15 genome obtained represents the first telomere-to-telomere (T2T) poplar genome.

Centromeres are specific regions on chromosomes where sister chromatids are cross-linked during cell division, ensuring their equal segregation during mitosis and meiosis^[52]^. They play a pivotal role in chromosome distribution. Notably, it is only recently that centromere sequences for specific plant species, such as Arabidopsis^[53]^, rice^[54]^ and maize^[55]^, have become available. However, our understanding of the sequences and structures of centromeres in poplar has remained limited. By employing quarTeT^[37]^, 19 centromeres were successfully identified within the DH15 genome, characterized by clusters of tandemly repeated sequences. These centromeres varied in length from 342,079 to 2,507,463 bp (Supplemental Table S10).

**Figure 3:**
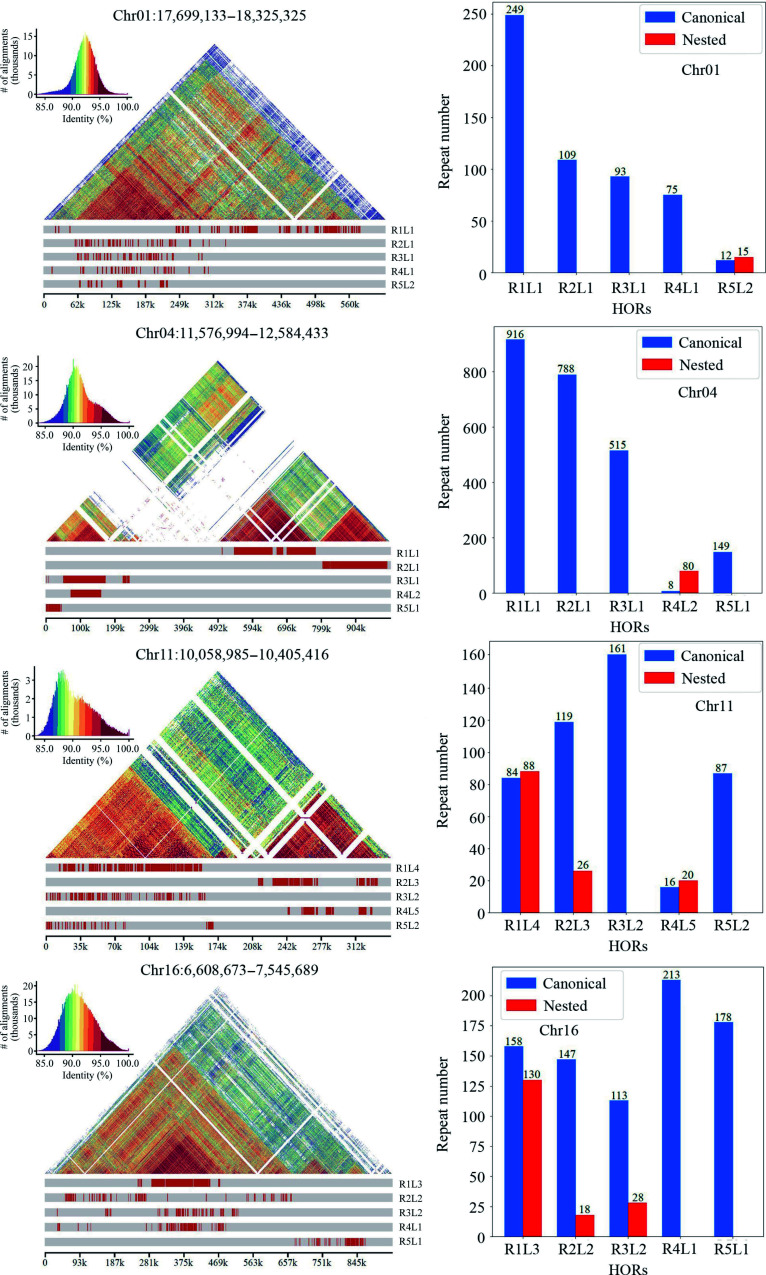
Number and distribution of each type of higher-order repeats (HORs) present in the centromere of each chromosome in the DH15 genome. Each type of HOR was denoted as 'R + rank of a HOR in the monomer pattern + L + the type of HOR units in a centromere'.

Annotation of centromeres, which includes the inference of monomers and the detection of higher-order repeats (HORs), is essential for studying the structure and evolution of centromeres within and between species^[56]^. In the current study, the 19 centromeres in the DH15 genome were annotated using HiCAT^[38]^. The top five monomers with the highest number of repeats on each chromosome were inferred and detected HORs (Fig. 3 & Supplemental Figs S3, S4).

**Figure 4:**
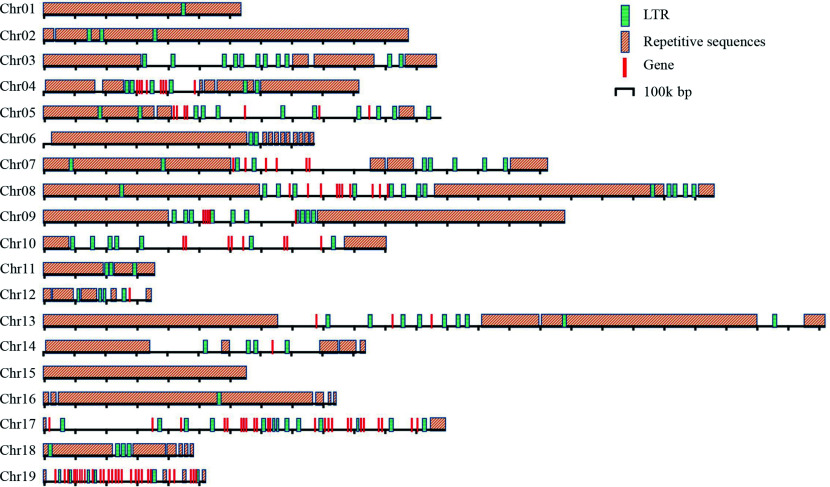
Distribution of the repetitive sequences, genes and LTRs in the centromeres of *P. ussuriensis*.

For Chr 1, HiCAT identified five frequent HORs, R1L1, R2L1, R3L1, R4L1 and R5L2. Each type of HOR was denoted as 'R + rank of a HOR in the monomer pattern + L + the types of HOR units in a centromere'. Notably, the R5L2 in Chr 1 is a combination of two HORs, featuring three monomers and two monomers, respectively. The locations of these HORs in all chromosomes were also analyzed (Fig. 3). The distribution of HORs can be classified into three types: (1) HORs spread all regions of centromeres. This type is exemplified by the centromeres of Chr 1, 2, 11, 15, and 16, all of which lack protein-coding genes (Supplemental Fig. S1); (2) HORs primarily cover the two ends of centromeres, leaving the central regions for protein-coding genes. These include the centromeres of Chr 3, 4, 5, 7, 8, 13, 14, 17 and 19, all of which except 3 harbor 8, 7, 6, 10, 3, 1, 24, and 28 protein-coding genes, respectively (Fig. 4). In the centromeres of Chr 17 and 19, the protein-coding genes extend to the two ends; (3) HORs are only distributed at one end of each centromere. These includes Chr 6, 9, and 12. Only 9 and 12 harbor 5 and 1 protein-coding genes in the central and one terminus, respectively.

Based on the centromere monomers from the 19 chromosomes of DH15, a phylogenetic tree was constructed. The present results revealed that all the DH15 centromere monomers could be divided into six distinct branches (Supplemental Fig. S5). The first branch included monomers from Chr 2, 6, 10, 11, 12, 13, and 15; the second branch included monomers from Chr 4, 5, 8, 14, 17, and 18; the third branch included monomers from Chr 3, 9, and 19, and finally the fourth, fifth, and sixth branches included monomers from Chr 7, Chr 16, and Chr1, respectively. Notably, the first and second branches held the lowest but equal hierarchy. In contrast, the hierarchies of the third to sixth branches increased gradually, indicating that the monomers from Chr 1 were evolutionarily most primitive.

The ratio of the longer arm to the shorter arm of each chromosome was then calculated based on the centromere location. Remarkably, the results (Supplemental Fig. S6, Supplemental Table S11) was in large agreement with the fluorescent *in-situ* hybridization (FISH) obtained in previous research^[57]^.

### Genome annotation

Annotation of coding genes in the DH15 genome was performed utilizing the EvidenceModeler pipeline, which combines *ab initio* predictions, homology-based searching and RNA-Seq data. In total, 34,953 protein-coding genes were identified. To assess the completeness and quality of the annotated proteome, the BUSCO protein model was used with the Embryophyta_odb10 database as a reference. The results indicated that 99.0% of the conserved proteins in BUSCO database were annotated in the DH15 genome, which accounts for 97.9% completeness and 1.1% fragment of BUSCO proteins (Supplemental Table S12). The BUSCO assessment indicated that the annotation of genome was of high accuracy.

The DH15 genome harbors a diverse array of repetitive elements, making up a substantial 43.18% of its total size, equivalent to 177.84 Mb. Within this, 30.41% (125.34 Mb) of the DH15 genome was specifically annotated as known repetitive elements, while 15.47% (63.77 Mb) remained unclassified. The most prevalent transposons in the DH15 genome were long-terminal repeats (LTRs), constituting a significant portion at 16.52%. Among the LTR elements, LTR/Gypsy occupied 7.57% (31.21 Mb), while LTR/Copia made up 6.16% (25.39 Mb) of the DH15 genome. DNA transposons, the second most abundant repetitive sequences, accounted for 6.16% (25.37Mb) of the DH15 genome. The remaining fraction consisted of LINES and Penelope elements (Supplemental Table S13).

**Figure 5:**
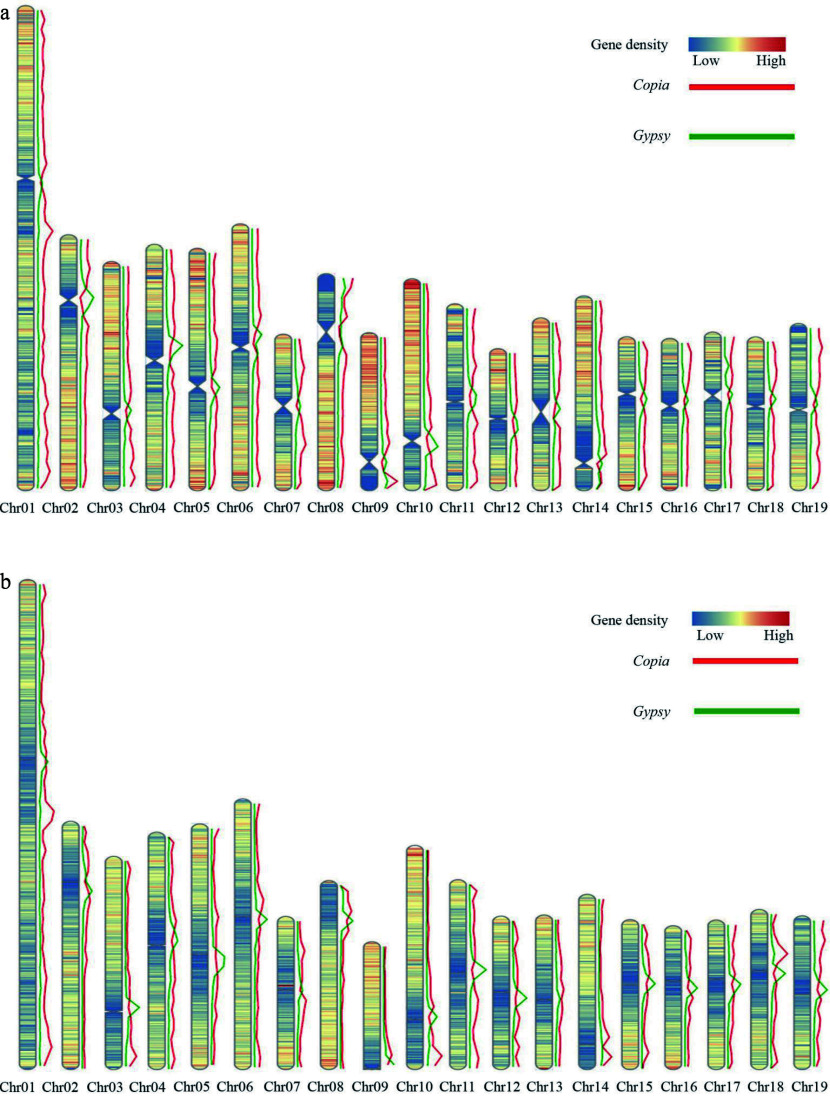
Distribution of gene density and elements on the DH15 chromosomes. (a) Distribution of gene density and distribution of *Copia* and *Gypsy* elements on the chromosomes of *P. ussuriensis*. (b) Distribution of gene density and distribution of *Copia* and *Gypsy* elements on the chromosomes of *P. trichocarpa*.

Further investigation of the distribution of *Gypsy* and *Copia* elements revealed that *Gypsy* and *Copia* elements were densely distributed in the regions with low protein coding gene density. The distribution of *Copia* elements were enriched in both ends of chromosome regions, and the distribution of *Gypsy* elements were enriched in the centromere regions of each chromosome (Fig. 5a). Gene density and *Gypsy*, *Copia* elements of *P. trichocarpa* were analyzed. A high degree of similarity in the distribution of gene density was found between the two genomes, with similar trends in the distribution of *Gypsy* and *Copia* elements (Fig. 5b).

### Genome evolution analysis

**Figure 6:**
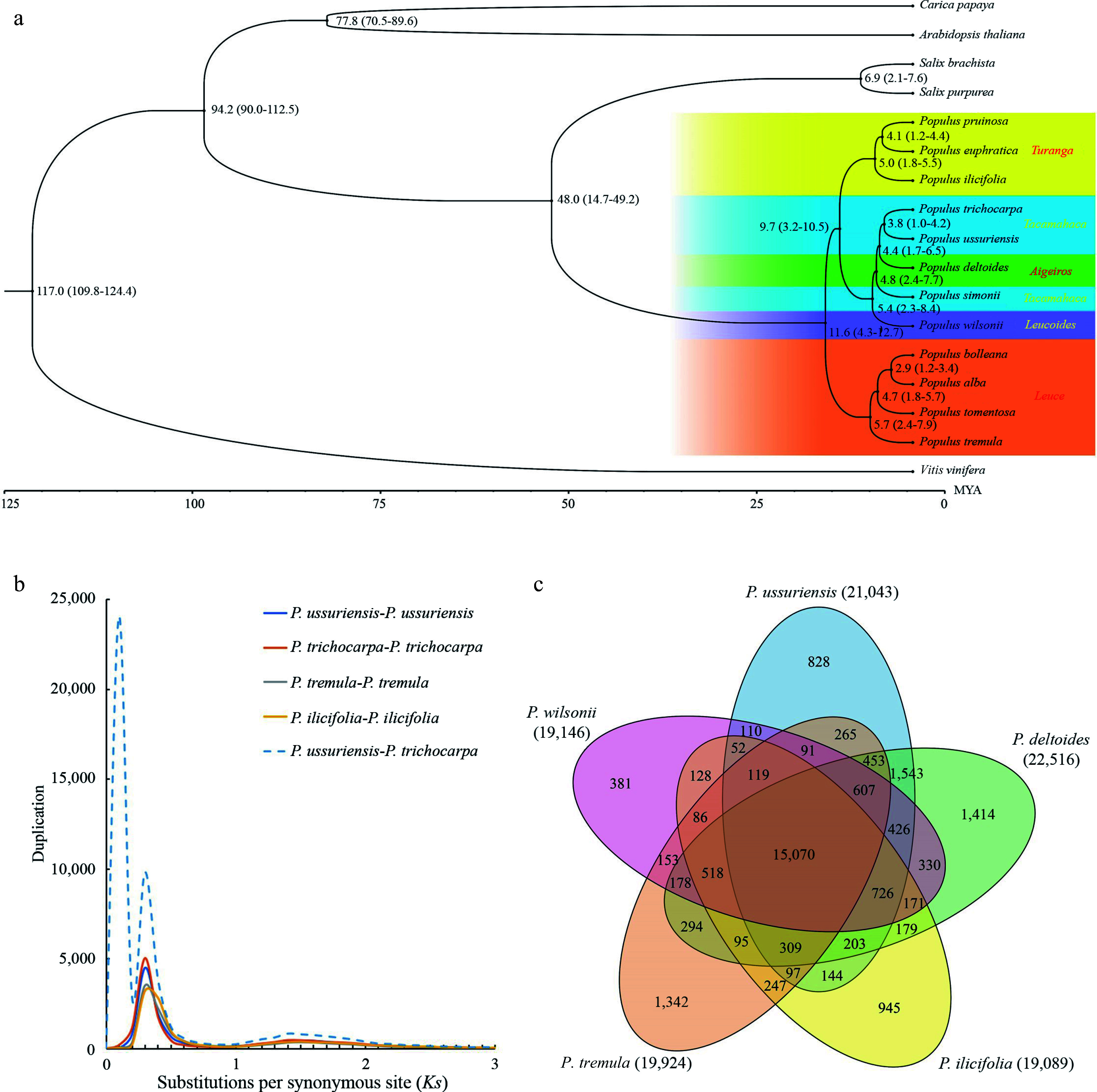
Evolutionary analysis of the *P. ussuriensis* genome. (a) Inferred phylogenetic tree of *P.ussuriensis* and 16 plant species based on protein sequences of single-copy orthologous genes. The numerical value beside each node is the estimated divergent time (million years ago, Mya) while the values in parentheses denote the range of the predicted divergence time). (b) Frequency distributions of synonymous substitutions (*Ks*) for the paralogous and orthologous genes within each of the following species: *P. ussuriensis*, *P. tremula*, *P. ilicifolia*, *P. trichocarpa*, and within each species. Additionally, we calculated *Ks* values between *P. ussuriensis* and *P. trichocarpa*. The lines with different colors represent the *Ks* distribution of different comparisons. (c) Venn diagram of the shared orthologous and paralogous gene families among five species: *P. ussuriensis*, *P. tremula*, *P. ilicifolia*, *P. deltoides* and *P. trichocarpa*.

To investigate the evolutionary trajectory, a comparative analysis of the DH15 genome was conducted against 16 plant species. Thirteen species in the Salicaceae family were selected, including two from the *Salix* genus, *Salix purpurea* and *S. brachista*, and eleven from *Populus*. The *Populus* species represented five sections within the genus: (1) *Leuce*, including *P. alba*, *P. bolleana*, *P. tomentosa* and *P. tremula*; (2) *Aigeiros*, only *P. deltoides*; (3) *Tacamahaca*, represented by *P. trichocarpa* and *P. simonii*; (4) *Leucoides*, only *P. wilsonii*; (5) *Turanga*, encompassing *P. euphratica*, *P. pruinose* and *P. ilicifolia*^[58]^. Furthermore, three additional species were included, *Arabidopsis thaliana,* *Carica papaya* and *Vitis vinifera*. *V. vinifera* was chosen as the outgroup due to its considerable genetic divergence from *P. ussuriensis*. To construct a phylogenetic tree encompassing all 17 species, the iqtree tool^[44]^ was used, using the single copy gene families identified by Orthofinder (Fig. 6a). As expected, all the *Populus* species studied formed a strongly supported monophyletic group. The species within *Leuce* and *Turanga* sections were found to cluster into two distinct clades. Conversely, the species from *Tacamahaca, Aigeiros* and *Leucoides* sections formed a clade, suggesting close phylogenetic relationships among these three sections. Notably, the *Aigeiros* (*P. deltoides* and *P. simonii*) was found to be phylogenetically closer to the *Tacamahaca* species (*P. trichocarpa* and *P. ussuriensis*) than to the *Leucoides* (*P. wilonii* only), suggesting the possibility of a monophyletic origin for the first two sections. The estimated divergence time between *P. ussuriensis* and *P. trichocarpa* was approximately 3.8 million years ago (Mya). In addition, the divergence between *Populus* and *Salix* was around 48.0 Mya (Fig. 6a), consistent with previous findings^[12]^.

The synonymous substitution rate (*Ks*) was used to estimate the whole genome duplication event (WGD) level and divergence event time of *P. ussuriensis*. By analyzing the *Ks* distribution, a WGD was inferred based on paralogous pairs and a species divergence event based on orthologous pairs (Fig. 6b). The distribution of *Ks* among syntenic genes of *P. ussuriensis* and *P. trichocarpa* displayed three peaks. One of these peaks, with a *Ks* range of 0.22−0.26, indicates a common WGD event that poplar species experienced. Such WGD events are well-documented occurrences in the evolution of angiosperms^[59,60]^. Another peak, centered around *Ks *= 0.01, signifies a recent divergence between *P. ussuriensis* and *P. trichocarpa* at the inter-species level. This finding is in agreement with the results obtained from the phylogenetic tree analysis (Fig. 6b).

Gene family analysis was also conducted on five selected species from different *Populus* sections: *P. ussuriensis*, *P. wilsonii*, *P. tremula*, *P. ilicifolia* and *P. deltoides*. The analysis revealed that the DH genome comprised 35,532 genes, organized into 21,043 gene families. These gene families exhibited some variations in size, with the largest family containing 61 genes. The specific and shared gene families among these five species were then investigated. Notably, 15,070 gene families were identified that were present in all the five species, while 828 gene families were specific to the lineage of *P. ussuriensis* (Fig. 6c). Subsequently, GO enrichment analysis was conducted for these gene family specific to the *P. ussuriensis*. This analysis revealed that these genes were primarily associated with functions related to 'phosphate-containing compound metabolic processes', This functional specialization may be related to the cold resistance of *P. ussuriensis*, as suggested in prior research^[61]^.

### Comparison of the DH15 genome to other *Populus* genomes

Comparative analysis of the DH15 genome was conducted in comparison to currently available poplar genomes. The results clearly demonstrated that the DH15 genome excelled in terms of contiguity and overall quality, as evident from the reduced gap numbers and the impressive N50 length (Supplemental Table S14). The current v4.1version of *P. trichocarpa* genome, which is known as the first fully sequenced tree species genome, stands out as highest quality among all the published poplar genomes, featuring 59 gaps and a contig N50 length of 13.16 Mb. Similarly, the *P. tremula* genome also exhibits relatively high quality, with 2,650 gaps and a contig N50 length of 1.16 Mb. In contrast, the DH15 genome assembled in this research contained seven gaps with contig N50 length of 19.50 Mb (Supplemental Table S14).

Telomere and centromere sequences are widely recognized as the most repetitive regions within a genome, which poses challenges for their assembly^[62]^. In the current *P. trichocarpa* genome, four chromosomes harbor telomere sequences at both ends, 14 have telomeres at one end, and one chromosome lacks telomeres at both ends. Similarly, in the *P. tremula* genome, two chromosomes harbor telomere sequences at both ends, ten chromosomes have telomere sequence at one end, and seven chromosomes lack telomeres at both ends. In contrast, all the 19 chromosomes of the DH15 genome contained telomeres at both ends.

**Figure 7:**
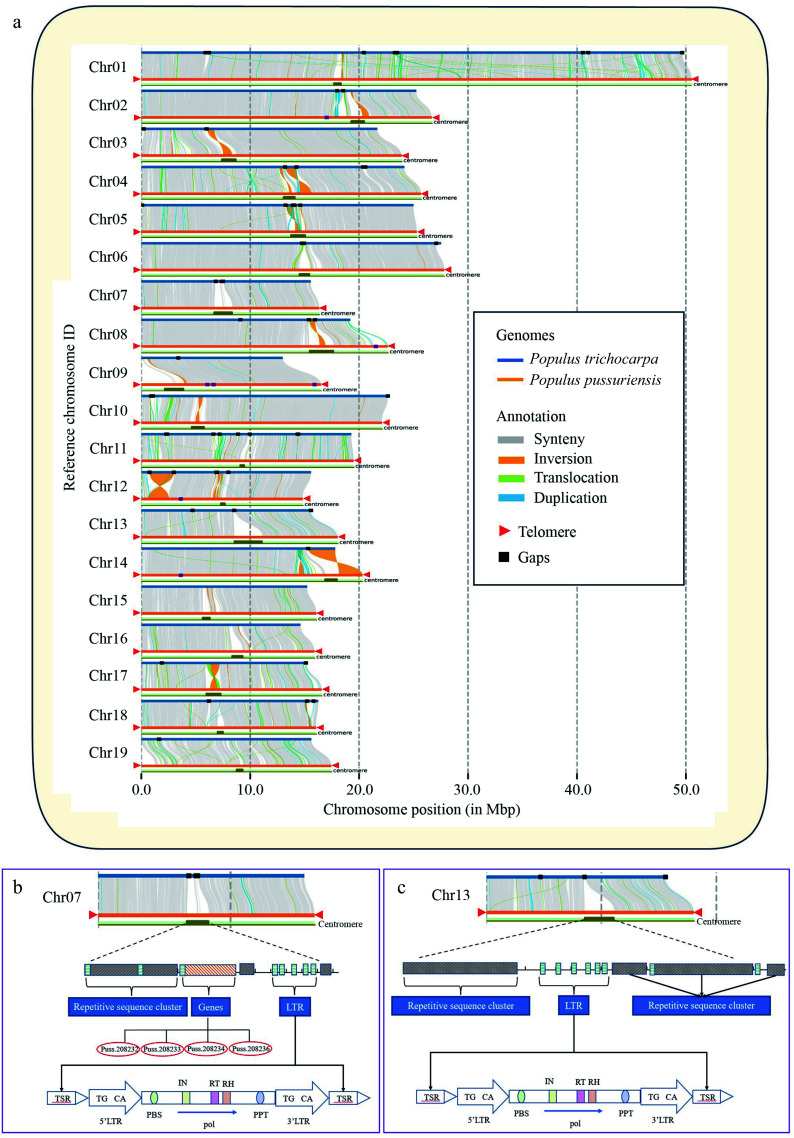
Genome collinearity, telomer and centromere structures in *P. ussuriensis* and *P. trichocarpa* genomes. (a) Collinearity between the genomes of *P. ussuriensis* and *P. trichocarpa* (gray lines). The red triangles mark the positions of telomere sequence repeats. The black rectangles illustrate the positions of gaps. (b) Structural delineation of *P. ussuriensis* Chromosome 07 centromere that corresponds to an unfilled gap in *P. trichocarpa* genome*.* (c) Structural delineation of *P. ussuriensis* Chromosome 13 centromere that corresponds to an unfilled gap in the *P. trichocarpa* genome.

quarTeT was employed to identify centromeres in the *P. trichocarpa* and the *P. tremula* genomes using the same parameter set as used for the DH15 genome. Compared with the *P. trichocarpa* and *P. tremula* genomes, the centromeres identified in the DH15 genome were more complete. In the *P. trichocarpa* genome, the centromeres vary in length, with the longest one located in Chr 3, spanning 230 kb, while the shortest, located in Chr 19, measured 30 kb. In the *P. tremula* genome, centromere lengths also vary, with the longest, in Chr 17, extending to 91 kb, and the shortest, in Chr 11, measuring just 3 kb. In contrast, the centromeres in Chr 3 and 19 of the DH15 genome were 1,258 kb and 536 kb, respectively, and the centromeres in Chr 17 and 11 were 1,296 kb and 346 kb, respectively. The total length of all centromeres was 1,612 kb in the *P. trichocarpa* genome, and 330 kb in the *P. tremula* genome. In striking contrast, the total length of all centromeres in the DH15 genome reached 20,978 kb (Supplemental Table S15). The collinearity analysis between the genomes of *P. trichocarpa* and DH15 unveiled that the gaps in the *P. trichocarpa* genome predominantly concentrated around centromere regions. Significantly, most of these missing sequences were successfully matched and assembled in the DH15 genome (Fig. 7a). For example, in the centromere regions of Chr 7, the *P. trichocarpa* genome contains three gaps, while the DH15 genome were contiguously assembled (Fig. 7b). In-depth analysis of Chr 7 in the DH15 genome revealed that within the centromeric region, there were tandem repetitions spanning a length of 0.86 Mb, constituting approximately 53% of the total centromere sequences on this chromosome. The rest was made up of LTR sequences and coding genes. These genes are found to be unique to DH15 and further confirmed by Polymerase Chain Reaction (PCR) (Supplemental Fig. S7). In the centromere region of Chr 13, it was worth noting that the *P. trichocarpa* genome exhibited one gap while the DH15 genome was contiguously assembled in this region (Fig. 7c).

The DH15 genome exceeds the length of the *P. trichocarpa* genome (v4.1) by 22.92 Mb. Consequently, the centromere and telomere sequences assembled in the DH15 genome are 19.89 Mb longer than those in the *P. trichocarpa* genome (as outlined in Supplemental Table S16). It is evident that these repetitive regions contribute significantly to the major difference in length between the two genomes. Protein-coding genes within the centromere regions in the DH15 genome were further annotated, revealing a total of 104 genes distributed across 11 centromere regions. To determine their presence in the *P. trichocarpa* genome, we conducted a blast-search of these genes against the *P. trichocarpa* proteins. Finally, 47 new genes in the centromere regions of the DH15 genome were identified. These genes were annotated using the NCBI and TAIR resources. Of them, 34 genes were functionally unknown and 13 were annotated as a MYB domain-containing gene, a photosystem II 44 kDa gene, and multiple CAP-Gly domain-containing linker genes, etc. (Supplemental Table S17). Transcriptome data was then used to investigate the expression levels of these centromeric genes and the transcripts of 23 genes were detectable.

## Discussion

The emergence of the next- and third-generations of DNA sequencing technologies, coupled with advanced bioinformatics tools, have greatly facilitated the sequencing, assembly, and public release of several poplar genomes^[9,10,14,63]^. Recent advancements in sequencing technologies, notably the widespread availability of highly accurate long-read sequencing provided by PacBio, along with the adoption of diverse assembly algorithms have significantly enhanced the quality of the published poplar genomes, particularly those published recently. However, the quest for achieving optimal genome contiguity and completeness remain a persistent challenge, especially when dealing with large, structurally diverse, hybrid, or heterozygous genomes. Notably, all published poplar genomes display incompleteness in their centromere and telomere regions, falling short of attaining a high level of contiguity. These problems have mainly arisen from two aspects: (1) poplars are dioecious plants whose genomes are often highly heterozygous^[1]^; (2) whole genome duplication, widespread events such as repetitive sequence expansions, and subsequent chromosome rearrangements have made poplar genomes more complicated and difficult to assemble. To solve these problems, a doubled haploid callus line of *P. ussuriensis *was generated, an ideal material for genome assembly. The DH15 genome represents a significant improvement over previously released poplar genome. It has successfully filled the majority of gaps, accomplished the closure of all telomeres and centromeres across its 19 chromosomes, and improved the representation of repetitive regions, including transposons, in comparison to the earlier poplar genome assemblies. Indeed, seven gaps in the DH15 assembly remain unclosed, and it's reasonable to suspect that these gaps consist of rDNA clusters. This assumption is supported by the annotation of the remaining 41 contigs, totaling 5.35 Mb in length, which revealed the prevalent presence of small subunit ribosomal RNA genes within these contigs, and they could not be assigned to specific chromosomes. The results indicated that the length of HiFi reads is insufficient to span the repeat regions of rRNA clusters. This is in line with findings in the human genome, where the majority of rRNA clusters are typically detected as 3 Mb DNA fragments^[64]^.

The near-gapless assembly of the *Arabidopsis thaliana* genome has enabled epigenomic profiling of centromeres and analysis of transposon insertion patterns^[53]^. In a similar vein, the identification and annotation of centromere regions of DH15 genome represent a crucial step toward conducting comparative sequence and epigenetic analyses of centromere evolution within the Populus genus, shedding light on its relation to speciation^[65]^. This high-resolution view of centromeric regions in DH15 offers a unique opportunity to investigate the origins and evolution of satellite repeats within centromeric regions. Moreover, it provides valuable insights into the organization and functioning of centromeres, not only within the poplar species but also in a broader biological context.

When comparing the DH15 genome to the *P. trichocarpa* genome, several notable improvements became evident. The DH15 genome successfully resolved many (> 50) assembly gaps present in the *P. trichocarpa* genome, this had a significant impact on gene prediction, resulting in more accurate and comprehensive gene annotations. Furthermore, the DH15 genome revealed a greater number of repeat sequences compared to the *P. trichocarpa* genome. Additionally, a karyotype analysis of the DH15 genome was performed and the results compared with previous experiments. The ratio of long arm to short arm of Chr 14 was quite different. In a previous study, the ratio of long and short arm was 3.23 in the *P. trichocarpa*, and in this study, the ratio of long and short arm was 5.48 in the DH15 genome. This discrepancy may be attributed, at least in part, to the presence of 45S rDNA. However, the ratios of long and short arms for other chromosomes remained relatively consistent, with only minor variations^[57]^.

## Conclusions

By utilizing a doubled haploid callus induced from an anther of a paternal tree and leveraging cutting-edge PacBio long-read sequencing technology, we successfully sequenced and assembled a nearly gapless, highly contiguous T2T *P. ussuriensis* genome. This achievement provides telomeric and centromeric composition and distribution, rendering it a valuable resource for various studies on poplar genomes. With this assembly, including high-resolution centromeric regions for all 19 chromosomes, we can significantly advance research on the evolutionary aspects of centromeres, their roles in shaping karyotypes, and their influence on speciation processes. Moreover, the new assembly creates opportunities for exploring the genetic and genomic functions of poplar centromeres, including their interactions with kinetochore proteins and their potential in the development of plant artificial chromosomes. Furthermore, it can expedite studies related to the generation of haploids and polyploids, thus advancing molecular breeding efforts. This study stands as a pivotal contribution to the field, offering indispensable genomic resources that will drive progresses in comparative genomics, genetic, and epigenetic studies, reproductive biology, and molecular breeding strategies for poplar trees.

## SUPPLEMENTARY DATA

Supplementary data to this article can be found online.

## Data Availability

The whole genome sequence data and the annotation in this article can be found in China National Center for Bioinformation (www.cncb.ac.cn) (ID: PRJCA017829).
