# Supplementary material for: A nearly gapless, highly contiguous reference genome for a doubled haploid line of Populus ussuriensis, enabling advanced genomic studies
Source: For Res (Fayettev). 2024 May 13;4:e019. doi: 10.48130/forres-0024-0016 (PMC11524312; doi:10.48130/forres-0024-0016)
Supplement: Supplementary file 1 — Supplementary data to this article can be found online. [file forres-0024-0016-S1.zip › 10.48130_forres-0024-0016-Suppl-FigureS1.pdf]

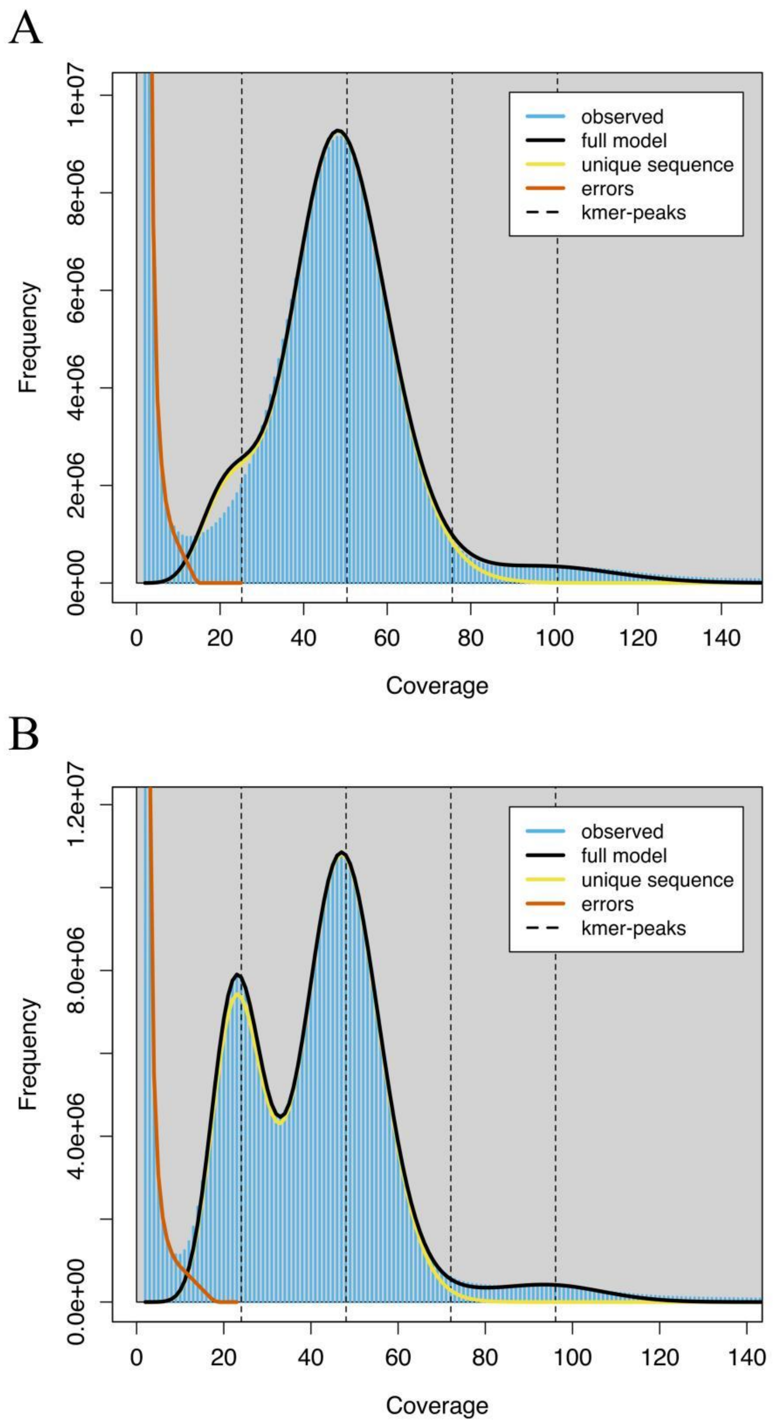

Supplementary Figure 1.
Estimation of genome size of *P. ussuriensis* based on K-mer analysis.
A. 21-mer frequency distribution of the DH15 homozygous callus line.
B. 21-mer frequency distribution of the paternal anther donor tree.
