# Supplementary material for: A nearly gapless, highly contiguous reference genome for a doubled haploid line of Populus ussuriensis, enabling advanced genomic studies
Source: For Res (Fayettev). 2024 May 13;4:e019. doi: 10.48130/forres-0024-0016 (PMC11524312; doi:10.48130/forres-0024-0016)
Supplement: Supplementary file 1 — Supplementary data to this article can be found online. [file forres-0024-0016-S1.zip › 10.48130_forres-0024-0016-Suppl-FigureS2.pdf]

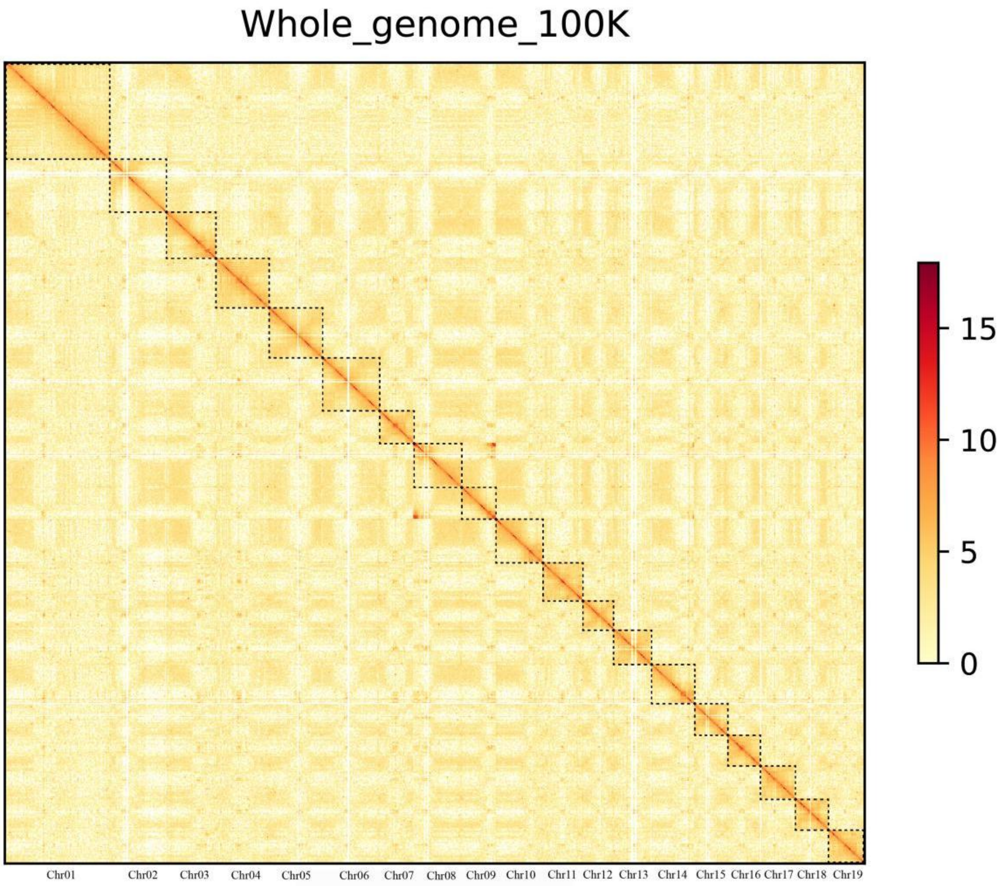

Supplementary Figure 2.
Heat map of Hi-C interactions based on chromosome-scale assembly.
