# Supplementary figures and images for: A nearly gapless, highly contiguous reference genome for a doubled haploid line of Populus ussuriensis, enabling advanced genomic studies
Source: For Res (Fayettev). 2024 May 13;4:e019. doi: 10.48130/forres-0024-0016 (PMC11524312; doi:10.48130/forres-0024-0016)

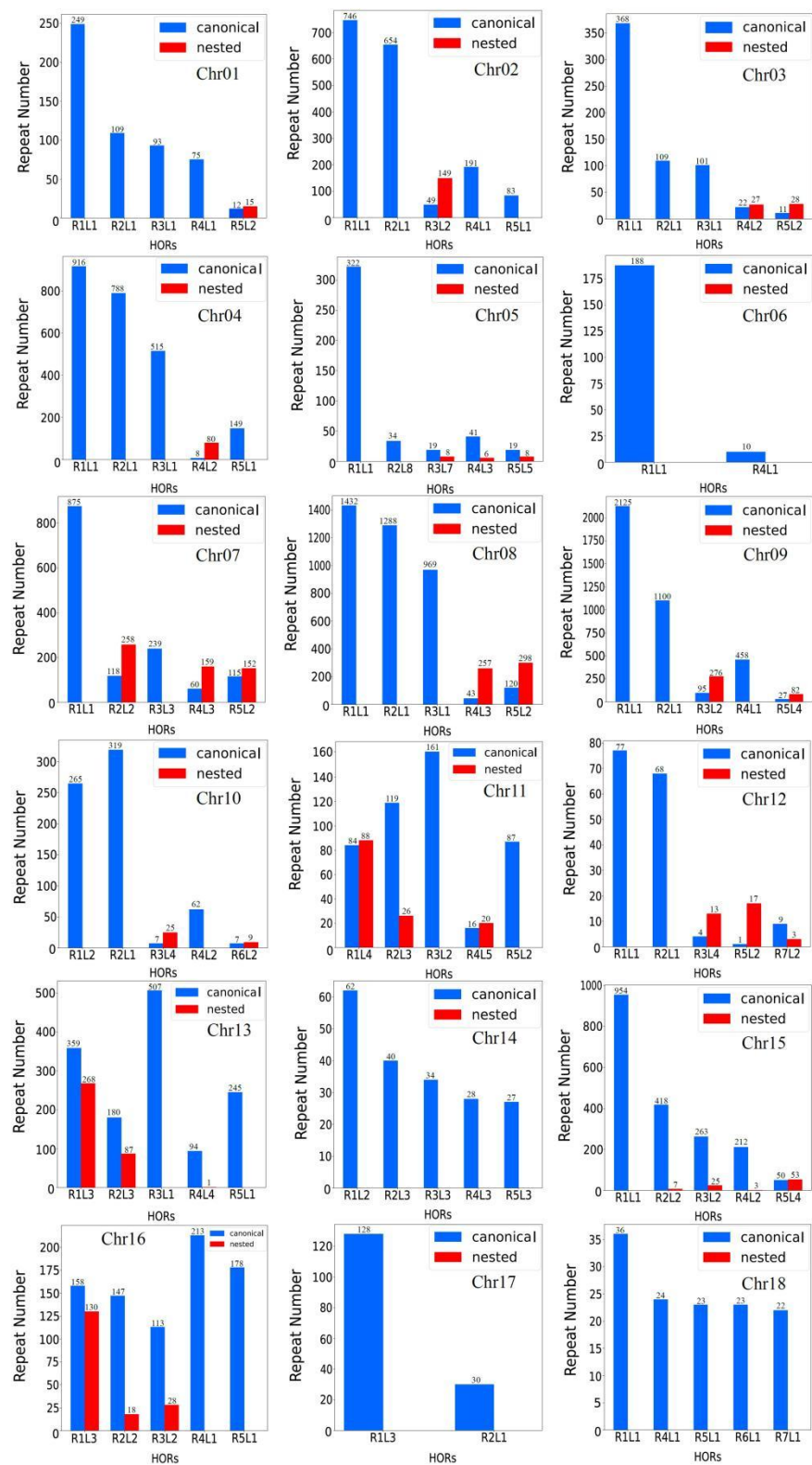

Supplementary Figure 3.

The numbers of HOR repeats in centromeres of 19 chromosomes.

Supplement: Supplementary file 1 — Supplementary data to this article can be found online. [file forres-0024-0016-S1.zip › 10.48130_forres-0024-0016-Suppl-FigureS3.pdf]

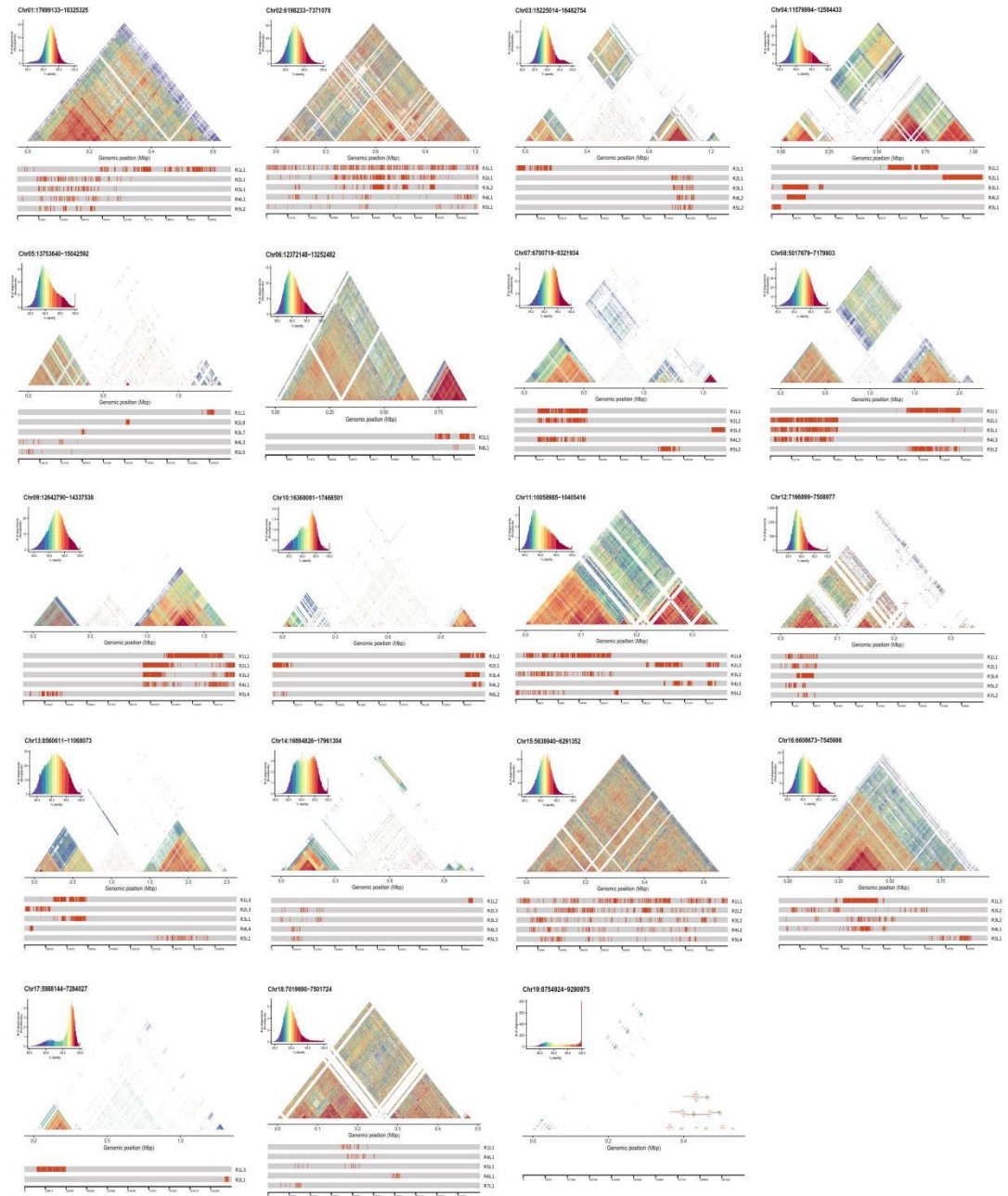

Supplementary Figure 4.  
Structure and annotation of 19 chromosome centromeres.

Supplement: Supplementary file 1 — Supplementary data to this article can be found online. [file forres-0024-0016-S1.zip › 10.48130_forres-0024-0016-Suppl-FigureS4.pdf]

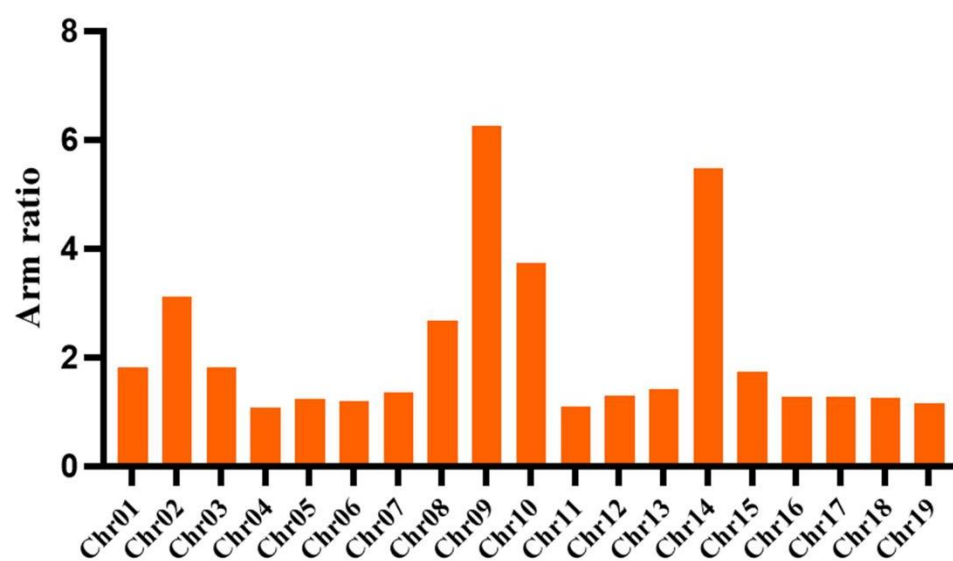

Supplementary Figure 6.

Arm ratio of chromosomes of *P. ussuriensis*.

Supplement: Supplementary file 1 — Supplementary data to this article can be found online. [file forres-0024-0016-S1.zip › 10.48130_forres-0024-0016-Suppl-FigureS6.pdf]

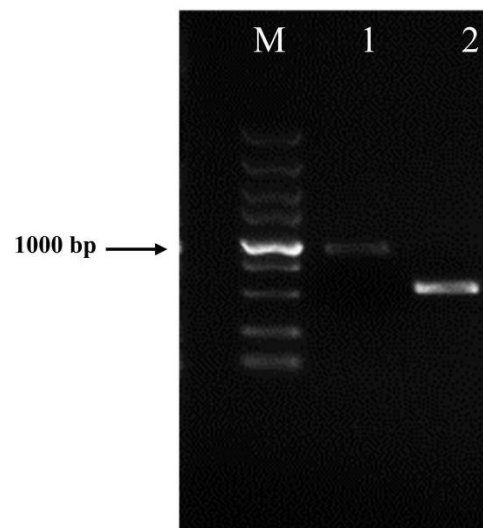

Supplementary Figure 7.

Specific primers were used to validate the genes. M: DL5000 marker. 1. Pus028233, 2. Pus028236.

Supplement: Supplementary file 1 — Supplementary data to this article can be found online. [file forres-0024-0016-S1.zip › 10.48130_forres-0024-0016-Suppl-FigureS7.pdf]
